# Case Report: Delayed diagnosis of adult-onset Still’s disease after sepsis-like acute liver failure with macrophage activation syndrome

**DOI:** 10.3389/fmed.2026.1941213

**Published:** 2026-10-13

**Authors:** Ruolan Quan, Changlin Wang, Wei Li, Kaiping Jiang, Kaizhou Huang

**Affiliations:** 1 The Eighth Clinical Medical College of Guangzhou University of Chinese Medicine, Foshan, China; 2 Foshan Hospital of Traditional Chinese Medicine, Foshan, China; 3 Department of Infectious Diseases, Foshan Hospital of Traditional Chinese Medicine, Foshan, China

**Keywords:** acute liver failure, adult-onset still's disease, macrophage activation syndrome, metagenomic next-generation sequencing, sepsis

## Abstract

**Background:**

Adult-onset Still's disease (AOSD) is a rare systemic autoinflammatory disease characterized by heterogeneous and nonspecific clinical manifestations. It often requires differentiation from infectious diseases, malignancies, and other rheumatic autoimmune disorders, making early diagnosis challenging. Acute liver failure (ALF) is a rare but severe complication of AOSD. AOSD may also be complicated by macrophage activation syndrome (MAS), a severe hyperinflammatory condition that can cause multiorgan dysfunction and mimic sepsis, further complicating diagnosis and management.

**Case presentation:**

We report a 35-year-old woman with AOSD complicated by macrophage activation syndrome who initially presented with acute liver failure. Multiple pathogens were detected by metagenomic next-generation sequencing (mNGS), leading to an initial misdiagnosis of sepsis. Over the following two years, the patient experienced recurrent episodes of fever, rash, arthralgia, and liver dysfunction. After comprehensive evaluation, the diagnosis of AOSD was established according to the Yamaguchi criteria, and MAS was diagnosed based on the HLH-2004 criteria and HScore. The patient achieved clinical remission after high-dose glucocorticoid therapy and remained stable during follow-up.

**Conclusions:**

This case highlights that AOSD complicated by MAS should be considered in patients with unexplained fever and acute liver failure, particularly those with hyperferritinemia, rash, and multisystem involvement. Even in the presence of evidence suggestive of infection, clinicians should remain vigilant for AOSD with MAS to avoid misdiagnosis and delayed treatment.

## 1 Introduction

Adult-onset Still's disease is a rare systemic autoinflammatory disorder characterized by heterogeneous clinical manifestations. Its etiology and pathogenesis remain incompletely understood, but it is generally considered to result from dysregulated immune responses and excessive systemic inflammation driven by a cytokine storm (1). Liver involvement is common in AOSD, occurring in approximately 81.3% of patients, although most cases present with mild-to-moderate liver enzyme elevations, whereas acute liver failure as a complication of AOSD is relatively uncommon (2). Macrophage activation syndrome (MAS) is one of the most severe complications of AOSD, with a reported incidence of approximately 5%–19.5%. It can lead to multiple organ dysfunction and represents a major contributor to mortality in affected patients (3). When MAS occurs, uncontrolled cytokine release and sustained hyperinflammatory responses may further aggravate hepatic injury and potentially result in acute liver failure (4).

Sepsis is a life-threatening condition characterized by infection-induced dysregulated host responses leading to organ dysfunction (5). During the acute active phase, particularly when complicated by MAS, AOSD may present with clinical manifestations and inflammatory profiles that closely resemble those of sepsis, including high fever, leukocytosis with neutrophilia, elevated levels of C-reactive protein (CRP) and procalcitonin (PCT), hepatic dysfunction, and coagulation abnormalities. These overlapping features make AOSD-associated MAS particularly challenging to distinguish from sepsis during the early stages of disease, often resulting in misdiagnosis (6).

This paper presents the case of a patient with adult-onset Still's disease (AOSD) complicated by macrophage activation syndrome (MAS) and presenting with acute liver failure, initially misdiagnosed as sepsis. This case highlights the diagnostic challenges in distinguishing AOSD-associated hyperinflammation from infection and emphasizes the importance of early recognition and treatment of MAS in AOSD patients.

## 2 Case presentation

A 35-year-old woman with a history of nephrotic syndrome had been receiving long-term oral prednisone (8 mg/day) maintenance therapy. In February 2023, she was admitted with a 1-day history of fever and abdominal pain. Laboratory tests at that time revealed marked hepatocellular injury [alanine aminotransferase [ALT] 1,116.6 U/L, aspartate aminotransferase [AST] 2,086.5 U/L, total bilirubin [TBIL] 62.4 μmol/L] and leukocytosis [white blood cell count (WBC) 23.54 × 10^9^/L, neutrophil count 19.73 × 10^9^/L]. An acute liver injury of unclear etiology was initially considered.

Further laboratory evaluation revealed coagulation abnormalities [prothrombin time [PT] 33.1 s, prothrombin activity [PTA] 21%, international normalized ratio [INR] 3.05], procalcitonin (PCT) 2.77 ng/mL, bicytopenia [hemoglobin (Hb) 89 g/L, platelet count 82 × 10^9^/L], and triglyceride (TG) 2.23 mmol/L. Metagenomic next-generation sequencing (mNGS) detected low abundance sequences of Pseudomonas aeruginosa (40 reads), Klebsiella pneumoniae (37 reads), and human herpesvirus 4 (40 reads). Testing showed no evidence of hepatitis A, B, C, or E virus infection. Tumor markers, including AFP, CEA, CA125, CA15-3, CA72-4, HE4, ROMA-P1, and ROMA-P2, were within the normal ranges. Autoimmune serological tests, including ANA, AMA-M2, LKM-1, GP210, LC-1, SLA/LP, SP100, PR3-IgG, MPO-IgG, and GBM-IgG, were negative. Blood cultures showed no significant abnormalities. Bone marrow biopsy revealed active hematopoiesis. Chest CT showed bilateral pulmonary inflammatory changes, focal consolidation in the lower lobes, and moderate pleural effusion. Abdominal CT revealed mild splenomegaly, enlarged lymph nodes in the hepatic hilar and retroperitoneal regions, and a small amount of ascites.

On day 1, empirical antimicrobial therapy with ceftriaxone sodium (2 g/day) was initiated, together with magnesium isoglycyrrhizinate (200 mg/day), polyene phosphatidylcholine (930 mg/day), and ademetionine (1 g/day). As her symptoms showed no significant improvement, antimicrobial therapy was escalated to imipenem/cilastatin (3 g/day) on day 3. Despite treatment, her condition continued to progress. On day 10, she developed altered mental status, characterized by inappropriate responses, disorientation, and impaired calculation ability, raising concern for hepatic encephalopathy. On day 11, erythematous patches developed over the trunk and extremities. Laboratory tests showed TBIL 362.5 μmol/L, LDH 395.3 U/L, PT 33.6 s, PTA 36%, INR 3.09, fibrinogen 1.47 g/L, and serum ferritin 10,278.04 ng/mL. Plasma exchange (PE) was performed on days 11, 13, and 15, with 3,000 mL of plasma exchanged during each session. Albumin (10 g/day) and L-ornithine L-aspartate (20 g/day) were administered concurrently. Methylprednisolone (80 mg/day; approximately 2 mg/kg/day) was subsequently initiated. Following treatment, her temperature gradually decreased, the erythematous patches resolved, and liver function gradually improved (Figure 1). She was discharged with diagnoses of acute liver failure and presumed sepsis.

**Figure 1:**
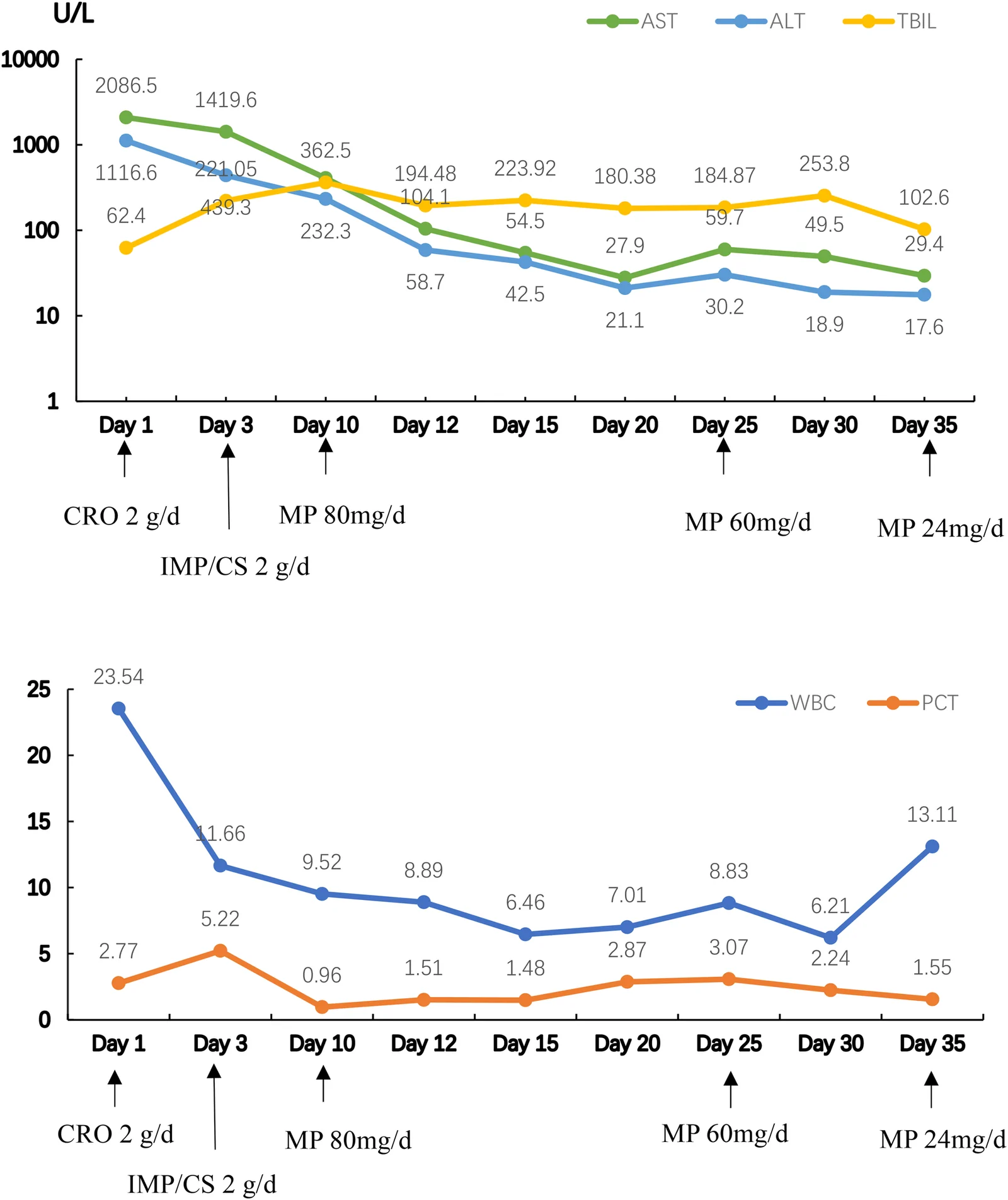
Schematic diagram of the trend of changes in treatment and laboratory test results. CRO, ceftriaxone sodium; IMP/CS,Imipenem/cilastatin 2 g/day; MP, methylprednisolone.

Between 2023 and 2025, the patient experienced recurrent fever (up to 39.5°C), accompanied by transient salmon-pink maculopapular rash, arthralgia, and liver dysfunction (peak ALT, 330.4 U/L; peak AST, 555.8 U/L). During these episodes, she received several courses of antimicrobial therapy, including levofloxacin (0.5 g/day), cefotaxime sodium (3 g/day), and meropenem (1 g/day), but showed limited clinical improvement. In contrast, glucocorticoid therapy at 1–2 mg/kg/day was associated with improvement in her clinical symptoms.

In February 2025, the patient was admitted with a 20-day history of fever (up to 39.6°C), accompanied by cervical lymphadenopathy, polyarthritis, and a diffuse pruritic erythematous rash. Laboratory tests revealed hyperferritinemia (8,121.36 ng/mL), an elevated erythrocyte sedimentation rate (ESR; 56 mm/h), bicytopenia (hemoglobin 89 g/L and platelet count 82 × 10^9^/L), hypertriglyceridemia (TG 3.45 mmol/L), hypofibrinogenemia (fibrinogen 1.45 g/L), and reduced NK-cell activity of 9.01%. PET/CT revealed mildly increased metabolic activity in multiple lymph nodes, diffuse bone marrow uptake, and reactive splenomegaly. Bone marrow examination demonstrated active granulopoiesis without dysplasia. Based on the Yamaguchi criteria (Table 1), she fulfilled the diagnostic criteria for AOSD and the HLH-2004 criteria for secondary MAS (7, 8), with retrospective HScore calculation further supporting a high probability of MAS (Tables 2, 3). After the diagnosis was established, the patient received intravenous methylprednisolone (80 mg/day) for 7 days, with subsequent clinical improvement. Methylprednisolone was then switched to oral administration at 72 mg/day and gradually tapered to prednisone 8 mg/day for maintenance. As of August 2026, she remained on oral prednisone 8 mg/day without recurrence of fever, arthralgia, or rash. At the last follow-up, blood cell counts, ferritin, liver function, triglycerides, and fibrinogen were within the normal ranges, with no evidence of MAS recurrence.

**Table 1:** The main test results at different stages during the patient's hospitalization.

Variable	Reference range	Feb 2023 (pre-GC)	Mar 2023 (post-GC)	Nov 2023 (Relapse 1)	Sep 2024 (Relapse 2)	Feb 2025 (pre-GC)	Mar 2025 (post-GC)
White-cell count (×10^9^/L)	3.50–9.50	23.54	13.11	12.19	10.79	13.70	7.51
Neutrophil percentage (NEUT%)	40%–75%	83.80	70.40	81.31	82.01	86.30	63.80
Hemoglobin (g/L)	115–150	85.00	80.00	89.00	82.00	78.00	98.00
Platelet count (10^9^/L)	125–350	98.00	158.00	242.00	202.00	89.00	189.00
Alanine aminotransferase (U/liter)	7–40	1,116.60	17.60	97.60	330.4	87.30	6.10
Aspartate aminotransferase (U/liter)	13–35	2,086.5	29.4	44.8	555.8	102.1	35.8
Bilirubin (μmol/L)							
Total	3.4–23.3	362.50	102.6	8.80	18.4	11.1	6.6
Direct	0.0–6.8	271.09	49.7	2.44	9.1	3.6	1.8
Lactate dehydrogenase (U/liter)	135–215	395.30	122.7	621.7	627.9	394.1	187.2
C-reactive protein (mg/liter)	≤10.00	117.83	6.51	35.89	77.08	118.39	2.56
Serum Ferritin (ng/mL)	21.8–274.7	10,278.04	9,428.50	/	/	8,121.36	5,849.42
NK Cell Activity (%)	≥15.11	/	/	/	/	9.01	156
Triglycerides (mmol/L)	<1.7	2.23	1.89	1.05	2.56	3.45	1.27
Fibrinogen (mg/dL)	200–400	147.00	260.00	246.00	453.00	2.41.00	319.00
Procalcitonin (ng/mL)	≤0.05	2.77	<0.05	0.12	0.31	1.54	0.17

**Table 2:** Yamaguchi diagnostic criteria.

Diagnostic requirements	Yamaguchi diagnostic criteria	Clinical evidence (Feb 2025)	Clinical evidence (Feb 2026)
Diagnostic requirement	≥5 criteria, including ≥2 major criteria, after exclusion of infection, malignancy, and rheumatic diseases	Fulfilled (6 criteria: 3 major and 3 minor criteria)	Fulfilled (7 criteria: 4 major and 3 minor criteria)
Major criteria	1. Fever ≥ 39°C lasting ≥ 1 week	Onset: Feb 6, 2025; Tmax: 40.2°C; duration: 2 weeks	Onset: Jan 20, 2026; Tmax: 39.6°C; duration: 3 weeks
	2. Arthralgia lasting ≥ 2 weeks	Not observed	Onset: Jan 15, 2026; duration: 4 weeks
	3. Typical salmon-colored rash	Onset: Feb 17,2025; transient during febrile episodes	Onset: Jan 20, 2026; transient during febrile episodes
	4. Leukocytosis ≥ 10 × 10^9^/L with neutrophils ≥ 80%	WBC: 23.54 × 10^9^/L; neutrophils: 83.8%	WBC: 81.07 × 10^9^/L; neutrophils: 89.6%
Minor criteria	1. Sore throat	Not observed	Not observed
	2.Lymphadenopathy and/or splenomegaly	Splenomegaly	Splenomegaly
	3. Abnormal liver function tests	ALT 1,116.6 U/L; AST 2,086.5 U/L	ALT 103.4 U/L; AST 7.3 U/L
	4.Negative ANA and RF	Negative	Negative
Exclusion criteria	Infection, malignancy, and other rheumatic diseases	Alternative diagnoses not fully excluded	Alternative diagnoses adequately excluded

**Table 3a:** Hemophagocytic lymphohistiocytosis-2004 (HLH-2004) diagnostic criteria.

HLH-2004 criteria	Feb 2025	Feb 2026
Fulfillment of at least 5 of the 8 criteria	Fulfilled (5/8 criteria)	Fulfilled (6/8 criteria)
1. Fever ≥ 38.5°C	Fulfilled	Fulfilled
2.Splenomegaly	Fulfilled	Fulfilled
3.Cytopenias affecting ≥ 2 of 3 lineages	Fulfilled	Fulfilled
4.Hypertriglyceridemia and/or hypofibrinogenemia	Fulfilled	Fulfilled
5.Ferritin ≥ 500 ng/mL	Fulfilled	Fulfilled
6.Reduced or absent NK-cell activity	Not evaluated	Fulfilled
7. Hemophagocytosis in bone marrow, spleen, lymph node, or liver	Not observed	Not observed
8.Soluble CD25 (sIL-2 receptor) ≥2,400 U/mL	Not evaluated	Not evaluated

**Table 3b:** Hscore of the patients at admission.

Parameter	No. of points (criteria for scoring)	Feb 2023 (pre-GC)	Mar 2023 (post-GC)	Feb 2025 (pre-GC)	Mar 2025 (post-GC)
Known underlying immunosuppression	0 (no) or 18 (yes)	18	18	18	18
Temperature (°C)	0 (<38.4), 33 (38.4–39.4), or 49(>39.4)	49	0	33	0
Organomegaly	0(no),23(hepatomegaly or splenomegaly), or 38(hepatomegaly and splenomegaly)	23	23	23	23
No. of cytopeniast	0 (1 lineage), 24 (2 lineages), or 34 (3 lineages)	24	0	24	0
Ferritin (μg/L)	0(<2,000), 35 (2,000–6,000), or 50(>6,000)	50	50	50	35
Triglyceride (mmol/L)	0 (<1.5), 44 (1.5–4), or 64 (>4)	44	44	44	0
Fibrinogen (g/L)	0 (>2.5) or 30 (≤2.5)	30	0	30	0
Aspartate aminotransferase (U/L)	0 (<30) or 19 (≥30)	19	0	19	0
Hemophagocytosis on bone	0 (no) or 35 (yes) marrow aspirate	0	0	0	0
HScore		257	135	241	76

## 3 Discussion

Adult-onset Still's disease (AOSD) lacks specific diagnostic biomarkers, and its clinical manifestations substantially overlap with those of infectious diseases, malignancies, and other rheumatic diseases. In this case, the imaging findings and negative autoantibody tests did not support malignancy or other autoimmune diseases. The main diagnostic challenge was distinguishing active AOSD from infection. At disease onset, the patient presented with persistent high fever, severe liver injury, marked leukocytosis and neutrophilia, elevated procalcitonin (PCT) levels, pathogen sequences detected by metagenomic next-generation sequencing (mNGS), and bilateral inflammatory changes on chest CT. Sepsis was therefore initially considered, consistent with the initial clinical presentation. However, two sets of blood cultures and routine microbiological tests failed to identify a definite pathogen, and no further evidence of active infection was found. Moreover, repeated antimicrobial therapy produced limited clinical benefit, whereas glucocorticoid treatment resulted in rapid clinical improvement, suggesting that the acute liver failure was more likely associated with AOSD-related systemic hyperinflammation.

PCT may be elevated in AOSD even in the absence of infection, and elevated PCT levels have been associated with an increased risk of macrophage activation syndrome (MAS), potentially serving as an early warning marker of non-infectious inflammatory activity and MAS (9). Marked hyperferritinemia is frequently observed in AOSD and may support the diagnosis and assessment of disease activity (10).

It is noteworthy that although mNGS has high sensitivity for pathogen detection, the identification of microbial nucleic acids does not necessarily indicate active infection (11). In patients with AOSD complicated by MAS, immune activation may predispose to pathogen reactivation; therefore, mNGS positivity should be interpreted cautiously, as infection may serve as a trigger rather than definitive evidence of sepsis (12). In this case, the positive mNGS result initially supported the diagnosis of sepsis but was ultimately considered a confounding factor in the diagnostic process.

In February 2023, during the patient's initial disease onset, MAS had not yet been clinically diagnosed. However, retrospective assessment using the HLH-2004 criteria and the elevated HScore suggested that MAS may have already been present at that time. Furthermore, the HLH-2004 criteria have certain limitations in diagnosing adult rheumatic disease-associated MAS. Therefore, the HLH-2004 criteria should be combined with clinical manifestations, alongside scoring tools such as the HScore or MS Score, for a comprehensive evaluation. Nevertheless, these tools cannot completely differentiate MAS from diseases with overlapping features, such as severe infections or hematological malignancies (13, 14).

Another noteworthy feature of this case was the patient's long-term exposure to glucocorticoids. The patient had received low-dose methylprednisolone maintenance therapy for nephrotic syndrome for several years. Despite this treatment, she subsequently developed recurrent fever and severe systemic inflammation, eventually progressing to acute liver failure. This suggests that low-dose glucocorticoids may partially suppress inflammatory activity but may be insufficient to adequately control the systemic inflammation associated with AOSD (15). Moreover, long-term glucocorticoid exposure may increase infection susceptibility, further complicating the differentiation between infection and disease activity (16). Glucocorticoids remain a cornerstone of therapy for severe AOSD and MAS, while targeted immunomodulatory therapies, such as IL-1 or IL-6 inhibitors, may be considered in refractory or severe cases to achieve rapid control of hyperinflammation and prevent organ damage (1, 17).

## 4 Conclusion

This case highlights that AOSD should be considered in the differential diagnosis of patients presenting with unexplained fever and acute liver failure, particularly when accompanied by hyperferritinemia, evanescent rash, and multisystem involvement. Importantly, elevated PCT levels or positive mNGS results should not exclude the possibility of AOSD, as these findings may also occur in the context of systemic inflammatory activation. Long-term low-dose glucocorticoid therapy may be insufficient to prevent severe inflammatory flares, and more intensive anti-inflammatory treatment may be required in cases of increased disease activity or the development of MAS. This case emphasizes the need to recognize the inflammatory basis of sepsis-like presentations in AOSD, interpret ancillary diagnostic tests cautiously, and facilitate early diagnosis and appropriate treatment.

## Data Availability

The datasets presented in this study can be found in online repositories. The names of the repository/repositories and accession number(s) can be found in the article/Supplementary Material.
